# Respiratory acclimatization and psychomotor performance after rapid ascent and during 3 weeks at 3,100 m–A prospective cohort study in healthy individuals

**DOI:** 10.3389/fphys.2025.1530426

**Published:** 2025-03-03

**Authors:** Lara Muralt, Mona Lichtblau, Sayaka S. Aeschbacher, Maya Bisang, Kay von Gruenigen, Talant M. Sooronbaev, Silvia Ulrich, Konrad E. Bloch, Michael Furian

**Affiliations:** ^1^ Pulmonary Division and Sleep Disorders Center, University Hospital of Zurich, Zurich, Switzerland; ^2^ Swiss-Kyrgyz High Altitude Medicine and Research Initiative, Zurich, Switzerland; ^3^ National Center for Cardiology and Internal Medicine, Bishkek, Kyrgyzstan; ^4^ Research Department, Swiss University of Traditional Chinese Medicine, Bad Zurzach, Switzerland

**Keywords:** high altitude (MeSH), acclimatization, sleep-disordered breathing (SDB), postural control (MeSH), cognitive performance

## Abstract

**Background:**

Acclimatization to high altitudes over several weeks has not been extensively studied. Repeated physiological assessments were performed in healthy lowlanders staying at 3,100 m for 3 weeks. We hypothesized that acute exposure to 3,100 m results in hypoxemia, sleep-disordered breathing, and postural instability, while a 3-week acclimatization at 3,100 m will improve these outcomes.

**Methods:**

Sixteen healthy volunteers (23–33 years) underwent nocturnal pulse oximetry and nasal airflow monitoring during 1 night in Bishkek (760 m), and during nights 1, 8 and 22 at Too-Ashu (3,100 m), Kyrgyzstan. On each day after monitoring, reaction time [psychomotor vigilance test reaction time test, (PVT)] and postural control [center of gravity path length on balance board (COPL)] were assessed.

**Results:**

Compared to 760 m, mean nocturnal SpO_2_ dropped in the first night at 3,100 m from mean ± SD 94.8% ± 1.9% to 86.3% ± 2.9% and recovered partially to 89.8% ± 1.5% after 3 weeks (P < 0.05 both comparisons to 760 m). Corresponding median (quartiles) oxygen desaturation indices were 1.0/h (0.3; 2.2), 6.5/h (4.5; 12.1) and 6.4/h (4.2; 11.1) time in bed (P < 0.05 both comparisons to 760 m). Median (quartiles) reaction times were 226 ms (212; 231), 236 ms (210; 259) and 228 ms (212; 246), P = NS, all comparisons. COPL worsened from 25.1 ± 4.1cm to 27.1 ± 4.1 cm (P < 0.05) and 26.4 ± 3.7 cm (P = NS compared to 760 m).

**Conclusion:**

In healthy lowlanders staying at 3,100 m, nocturnal SpO_2_ increased over 3 weeks after an initial drop but did not reach baseline values. Postural control was impaired in the first week of acute exposure to high altitude despite improvements in hypoxemia. Altitude exposure did not affect reaction time. Thus, acute and prolonged exposure to hypobaric hypoxia has differential effects on oxygenation, control of breathing, postural control, and reaction time.

## Introduction

Mountain tourism accounts for 15%–20% of the annual global tourism revenue (approximately 296 billion US dollars in 2019) (Lock, 2020), highlighting the popularity of trips to mountainous regions, primarily to high altitude regions between 2,000 and 3,500 m, where the majority of mountain settlements and touristic places are located (i.e., La Paz, Bolivia situated at 3,640 m and being the residence of >800,000 inhabitants). However, high-altitude excursions expose the human body to a lower barometric pressure and reduced arterial blood oxygenation (hypoxemia), which requires numerous physiological adaptations to protect the body and organs against hypoxemia-related dysfunction and damage. However, already moderate acute hypoxemia can trigger the development of acute mountain sickness (AMS) (Bartsch and Swenson, 2013) and other conditions that compromise a stay at altitude, e.g., sleep-disordered breathing (Tseng et al., 2015), postural instability (Mutschler et al., 2023) and cognitive impairment (Reiser et al., 2023). These acute impairments have been investigated in detail, while less attention has been given to the subsequent acclimatization to high altitude.

The few publications investigating a stay at altitude for several weeks have, e.g., shown that the arterial oxygen saturation (SpO_2_) acutely decreases (Forrer et al., 2023) with altitude and redirects towards sea level values within weeks (Lundby et al., 2004; Bloch et al., 2010; Yan, 2014). Furthermore, sleep-disordered breathing (SDB) can occur, often caused by high altitude periodic breathing (Bloch et al., 2015; Weil, 2004). However, it is uncertain if SDB, which might impair subjective sleep quality, increases (Bloch et al., 2010), decreases (White et al., 1985), or remains unaffected (Zieliński et al., 2000) during acclimatization. At altitudes between 3,750 and 6,850 m, it has been observed that SDB increased during acclimatization over a period of more than 2 weeks despite an improvement in SpO_2_. Whether similarly prolonged ventilatory acclimatization takes place at altitudes of 3,000–4,000 m, which has a higher relevance for most travelers and workers, remains open.

Additionally, acute exposure to hypoxia leads to impairments in postural control (Mutschler et al., 2023; Stadelmann et al., 2015; Wagner et al., 2011; Cymerman et al., 2001; Nordahl et al., 1998) and cognitive performance at higher altitudes (Reiser et al., 2023; Yan, 2014; Hu et al., 2016; Tesler et al., 2015; Pun et al., 2019; Pun et al., 2018). However, the literature on cognitive and psychomotor functions at high altitudes remains conflicting–some reports indicate impairments, especially at very high altitudes (Yan, 2014; de Aquino Lemos et al., 2012; Kramer et al., 1993), others found no change (Luks et al., 1998; Latshang et al., 2013; Beaumont et al., 2007). It remains unknown whether and to which extent postural control and cognitive performance are impaired at 3,100 m and if they can recover during a few weeks of exposure to high altitude.

The current study aims to close gaps in knowledge of nocturnal breathing, psychomotor performance, and postural control during acute exposure to 3,100 m and subsequent acclimatization over 3 weeks. Understanding the underlying physiological acclimatization effects may help to prevent altitude-induced accidents, mental and physical impairments, and to improve wellbeing at altitude.

We hypothesized that acute exposure to hypobaric hypoxia at 3,100 m is associated with hypoxemia and SDB, which lead to impaired cognitive performance and postural control. Furthermore, we hypothesized that during a 3-week acclimatization period, SpO_2_, SDB, cognitive performance, and postural control would improve.

## Methods

### Study design and setting

Baseline evaluations of this observational study were carried out in Bishkek (760 m), Kyrgyzstan. Thereafter, participants were driven by minivan within 3 h to the Too-Ashu high-altitude clinic, located at 3,100 m. The assessments at 3,100 m were performed during the 1^st^, the 8^th^ and the 22^nd^ night and the following days (day 2, 9 and 23).

### Participants

Healthy lowlanders, 20–75 years of age, born, raised, and currently living below 1,000 m were recruited. Participants with any disease that requires treatment, as well as heavy smokers (>20 cigarettes per day) were excluded. The study was approved by the Ethics Committee of the National Center of Cardiology and Internal Medicine, Bishkek, Kyrgyzstan (01–8/405) and all participants gave informed written consent.

### Measurements

#### Nocturnal pulse oximetry and nasal airflow study

Nocturnal pulse oximetry and nasal pressure swing assessments were measured from 23:00 to 06:00 by using the ApneaLink™ device (ResMed 9001 Spectrum Center Blvd, San Diego, CA 92123, United States), which has been validated in several studies for screening of SDB (Chen et al., 2009; Nigro et al., 2012). For the manual scoring, the AASM Chicago Criteria were used for the definitions of apnea, hypopnea, and oxygen desaturation: apnea was defined as a ≥90% decrease in nasal pressure swings *versus* baseline for ≥10 s; hypopnea was defined as a decrease in nasal pressure swings by ≥50% to <90% for ≥10 s; oxygen desaturation was defined as a ≥4% decrease in SpO_2_ (Thornton et al., 2012). The apnea-hypopnea index (AHI) and the oxygen desaturation index (ODI) were computed as number of events per hour of time in bed.

#### Postural control

**FIGURE 1:**
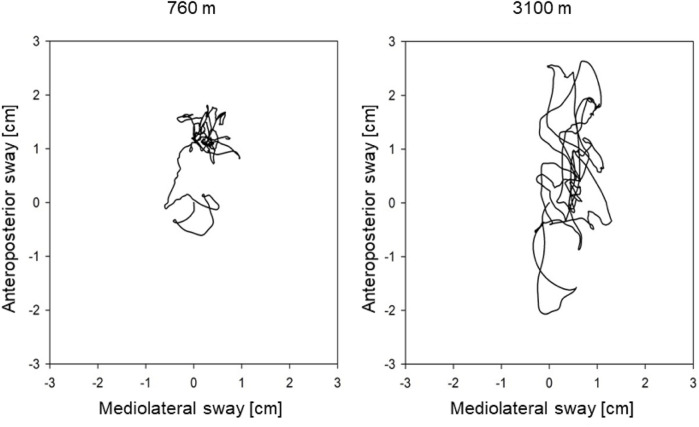
Example of a center of pressure path in an individual at 760 and 3,100 m.

Balance tests were performed on a Wii Balance Board™ (WBB), 30 × 50 cm in size (Wii Balance Board, Redmond, WA, United States). The WBB has four pressure sensors with a sample rate of 100 Hz to measure the center of pressure (COP). The longer the path (cm) of the COP is, the more the person swayed (see Figure 1). Besides the COP path length (COPL), the maximal amplitude and the sway velocity were measured in the anteroposterior and mediolateral axes.

The balance board was positioned 1.5 m in front of a wall. The participants stood on both legs with eyes open and feet in a 30° angle, as marked on the WBB. They were instructed to focus on a black dot on the wall at 1.7 m height, keep their hands beside the body, and remain as still as possible while performing tests of 30 s. The tests have been repeated 5 times and subsequently averaged.

A customized software (Labview 8.5, National Instruments, Austin, TX, United States) was used to track the COP and to analyze the data (Clark et al., 2010). The WBB was calibrated at each location and then once a week by placing a variety of known weights at different positions on the WBB. The assessment of the postural control using the WBB measurements has been validated by comparison with a laboratory-based force platform (Clark et al., 2010).

#### Psychomotor and cognitive performance

The 10-minute-long psychomotor vigilance test (PVT) is a sustained-attention, reaction-timed task test that measures cognitive performance (Alakuijala et al., 2014). Participants performed the PVT alone in a darkened room and were instructed to look at a little lamp on the device, which lighted up in irregular time intervals of 2–10 s. The participants had to press a button as fast as possible as soon as the light appeared (Alakuijala et al., 2014; Basner and Dinges, 2011). The trail-making test A (TMT-A), which requires connecting 90 encircled numbers by a line on a sheet of paper, was administered (Tombaugh, 2004). Participants performed two TMT-A versions at each testing session in random order, of which subsequently the mean was calculated.

#### Questionnaires and clinical examinations

In the mornings after the sleep studies, subjective sleep quality was assessed on a 100-mm visual analog scale labeled at 0 mm with “very bad, not slept at all” and at 100 mm with “very good, best sleep ever”. On this 100-mm horizontal line, participants marked a point that best represented their subjective sleep quality of the last night. Additionally, participants indicated the subjective time until falling asleep in minutes, as well as the number and duration of awakenings during the past night.

AMS was assessed using the Lake Louise questionnaire (LLQ) and the Environmental Symptoms Questionnaire cerebral score (AMSc). AMS was defined as having a LLQ score of >2 points including headache or an AMSc ≥0.7 during the first 2 days at 3,100 m (Roach et al., 2018; Sampson et al., 1983). Moreover, daytime SpO_2_ was assessed by finger pulse oximetry.

#### Outcomes and sample size estimation

The primary outcome was the difference in the mean nocturnal SpO_2_ (nSpO_2_) between night 1 and night 8 at 3,100 m measured by pulse oximetry. Secondary outcomes were additional changes in nSpO_2_ of night 22 compared to night 1 at 3,100 m. Additionally, changes in postural control, cognitive performance, and sleep patterns due to the ascent from 760 to 3,100 m and the subsequent 3 weeks of acclimatization were assessed. The sample size calculation was based on paired comparisons and a minimal important change in nSpO_2_ of 3% between night 1 and night 8 at 3,100 m with an SD of 3% (Latshang et al., 2013; Nussbaumer-Ochsner et al., 2012). To achieve a power of 80% with a significance level of 0.05 and accounting for a dropout rate of 20%, the required sample size was estimated to be 12 participants.

### Data analysis and statistics

All participants with SpO_2_ measurements of more than 3 hours during the four testing nights were included in the analysis. For the statistical analysis, the software STATA/SE 13.1 was used. Data was tested for normality by the Shapiro-Wilk-test. Depending on data distribution, the effect of altitude and time at altitude were evaluated using non-parametric Friedman analysis of variance (ANOVA) followed by Wilcoxon matched pairs tests, or for parametric data, repeated measures ANOVA followed by paired t-tests. Post-hoc tests were only performed when the ANOVA showed a significant result.

The influence of independent parameters like the time at altitude, nSpO_2_, age, sex, body height, or body mass index on the COPL and the ODI has been evaluated with multivariate regression analysis. To obtain parametric data, the ODI had to be logarithmically transformed. The trial was registered under www.clinicaltrials.gov, NCT02451020.

## Results

### Subjects

A total of 20 participants were invited. Of those, three were excluded at the beginning of the study due to lack of time, and one lost the nasal cannula for several nights and was therefore not included in the nocturnal respiratory analysis. Thus, nine males and seven females were included in the analysis (Table 1). One person fulfilled the AMS criteria on the second day at altitude with an LLQ score of 3 points. The mean scores for the LLQ and AMSc on the second day at 3,100 m were 0.8 ± 1.1 and 0.14 ± 0.13, respectively.

**TABLE 1:** Patient characteristics at 760 m.

Number (% female)	16 (43.8%)
Nationality, Kyrgyz/Swiss	7/9
Age, years	27 ± 3
Body weight, kg	71.4 ± 9.6
Body height, cm	173.8 ± 8.5
Body Mass Index, kg/m^2^	23.7 ± 3.2
FEV_1_, % predicted	97.8 ± 15.4
Arterial oxygen saturation, %	97.5 ± 0.7

Data is shown in absolute numbers or mean ± SD. FEV_1_, forced expiratory volume during the first second of expiration.

### Nocturnal pulse oximetry

**FIGURE 2:**
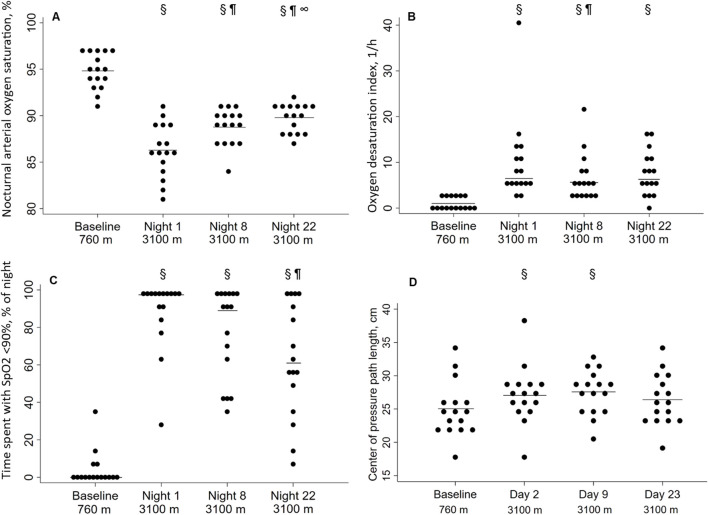
Effect of altitude and acclimatization. **(A)** Nocturnal arterial oxygen saturation during the sleep [%]; **(B)** Oxygen desaturation index [1/h]; **(C)** Time spent with SpO_2_ <90% [% of time in bed]; **(D)** Center of pressure path length [cm]. Each point represents a participant, the solid line represents the mean **(A, C)** or the median **(B, D)**. ^§^P < 0.05 vs. 760 m; ^¶^P < 0.05 vs. night 1 at 3,100 m; ^∞^P < 0.05 vs. night 8 at 3,100 m.

The results are presented in Table 2 and Figure 2. The median (quartiles) of the analyzed time in bed was 6.8 h (6.0; 7.5) and did not differ between the nights. On the first night at 3,100 m, the mean nSpO_2_ was decreased (94.8% ± 1.9% vs. 86.3% ± 2.9%), and time of nSpO_2_ <90% was increased (0.0% vs. 97.5% of time in bed) compared to baseline, but both improved partially over 3 weeks at altitude (Figures 2A,C). nSpO_2_ recovered from the first night at 3,100 m to the eighth night from 86.3% to 88.8% (2.9% increase) and from the eighth to the 22nd night from 88.8% to 89.8% (1.1% increase). Time of nSpO_2_ <90% recovered from 97.5% to 89.0% (8.7% decrease) and from 89.0% to 61.0% (31.5% decrease), respectively.

**TABLE 2:** Effect of altitude and acclimatization on nocturnal breathing and daytime performance.

	760 m	3,100 m (night 1/day 2)	3,100 m (night 8/day 9)	3,100 m (night 22/day 23)	P-value^a^
Time in bed, hours	6.7 (5.4; 6.9)	7.1 (6.1; 7.8)	6.8 (6.3; 7.3)	7.3 (6.3; 7.7)	0.466
Nocturnal SpO_2_, %	94.8 ± 1.9	86.3 ± 2.9^b^	88.8 ± 1.9^b^ ^,^ ^c^	89.8 ± 1.5^b^ ^,^ ^c^ ^,^ ^d^	<0.001
Oxygen Desaturation Index, events/hour	1.0 (0.3; 2.2)	6.5 (4.5; 12.1)^b^	5.7 (3.6; 8.7)^b^ ^,^ ^c^	6.4 (4.2; 11.1)^b^	<0.001
Sleep time <90% SpO_2_, % of time in bed	0.0 (0.0; 4.0)	97.5 (87.0; 100.0)^b^	89.0 (52.0; 96.5)^b^	61.0 (41.5; 94.0)^b^ ^,^ ^c^	<0.001
Sleep time <85% SpO_2_, % of time in bed	0.0 (0.0; 2.0)	36.0 (3.5; 76.5)^b^	1.0 (0.0; 7.5)^c^	1.0 (0.0; 1.5)^c^	<0.001
Nocturnal breathing frequency, or respiratory rate, events/min	17.0 ± 1.9	17.3 ± 1.7	16.9 ± 1.7	16.6 ± 2.1	0.316
Apnea-Hypopnea Index^e^, events/hour	5.6 (2.3; 7.8)	6.4 (3.5; 8.4)	6.2 (2.5; 8.6)	9.1 (2.3; 18.6)	0.249
Heart frequency, bpm	62 ± 6	68 ± 9^b^	70 ± 11^b^	66 ± 13	0.003
Awakenings, events	1 (0; 2)	3 (1; 3)^b^	1 (1; 2)^c^	1 (0; 3)^c^	0.034
Duration of awakenings, min	6 (0; 13)	11 (0; 50)^b^	5 (1; 10)	5 (0; 7)^c^	0.040
Subjective sleep quality^f^, %	70 (48; 77)	55 (27; 79)	73 (46; 84)	63 (45; 81)	0.460
Daytime SpO_2_, %	97.5 ± 0.7	94.0 ± 1.0^b^	93.9 ± 1.7^b^	94.2 ± 1.4^b^	<0.001
Median psychomotor vigilance test reaction time (PVT), ms	226 (212; 231)	236 (210; 259)	237 (212; 244)	228 (212; 246)	0.115
Trail Making Test or Trail making test (result?), s	55 (50; 61)	53 (45; 56)^b^	49 (40; 54)^b^ ^,^ ^c^	47 (42; 53)^b^ ^,^ ^c^	<0.001
Center of pressure path length, cm	25.1 ± 4.1	27.1 ± 4.1^b^	27.5 ± 3.2^b^	26.4 ± 3.7	0.048
Anteroposterior amplitude, cm	1.85 (1.46; 2.22)	1.82 (1.67; 2.13)	1.91 (1.62; 2.17)	1.63 (1.47; 2.33)	0.299
Anteroposterior velocity, cm/s	0.56 ± 0.11	0.64 ± 0.11	0.67 ± 0.09^b^	0.63 ± 0.14	0.027
Mediolateral amplitude, cm	1.36 (1.21; 1.56)	1.31 (1.21; 1.43)	1.32 (1.07; 1.50)	1.22 (1.04; 1.44)	0.131
Mediolateral velocity, cm/s	0.47 ± 0.10	0.51 ± 0.09	0.49 ± 0.10	0.48 ± 0.08	0.169

Nonparametric values are shown in median (interquartile range); parametric values in mean ± SD.

^a^ Overall effects of repeated measures analysis of variance or Friedman ANOVA as appropriate.

^b^ P < 0.05 vs. 760 m.

^c^ P < 0.05 vs. night 1 or day 2 at 3,100 m.

^d^ P < 0.05 vs. night 8 or day 9 at 3,100 m appraised with a paired t-test or Wilcoxon rank sum test as appropriate. SpO_2_, arterial oxygen saturation.

^e^ Apneas and hypopneas were defined via the AASM criteria (see method section for more information).

^f^ Subjective sleep quality was assessed by a 100-mm visual analogue scale, ranging from 0 “very bad” to 100 “very good”.

In contrast, the ODI remained unchanged for 3 weeks after the initial increase (Figure 2D). The regression analysis showed a positive correlation between altitude and ODI, even when adjusting for age, sex, and body mass index (Table 3). Body mass index was a significant positive predictor for a higher ODI, whereas female sex was associated with a lower ODI. Nocturnal breathing frequency did not change during the altitude sojourn, while the mean of nocturnal heart frequency increased significantly at altitude and remained elevated for at least 1 week.

**TABLE 3:** Multivariate linear regression in Log10 oxygen desaturation index [events/hour].

	Coefficient	95% CI	P-value
Time at altitude			
1st night at 3,100 m (Reference)			
Night at 760 m vs. 1st night at 3,100 m	−2.14	−2.73 to −1.55	<0.001
8th night at 3,100 m vs. 1st night at 3,100 m	−0.27	−0.48 to −0.06	0.012
22nd night at 3,100 m vs. 1st night at 3,100 m	−0.24	−0.64 to 0.16	0.238
Age, years	0.07	−0.01 to 0.14	0.083
Sex (female)	−0.56	−1.06 to −0.06	0.028
Body mass index, kg/m^2^	0.10	0.06 to 0.14	<0.001
Intercept	−1.21	−3.44 to 1.02	0.286

CI, confidence interval.

Subjective sleep quality at 3,100 m remained unchanged compared to 760 m, but the participants reported more awakenings during the first night at 3,100 m and estimated awakenings to be slightly longer.

### Postural control

With ascent from 760 m to 3,100 m, COPL worsened significantly (Table 2; Figure 2B), remained impaired on day 9, but recovered after 3 weeks at 3,100 m. The deterioration of postural control was mainly due to the change in the anteroposterior direction (Table 2). The regression analysis revealed a positive correlation of nSpO_2_ with altitude, and a negative correlation of nSpO2 with COPL during at least 2 weeks, and no correlation of COPL with age, sex, or body height (Table 4).

**TABLE 4:** Multivariate linear regression in center of pressure path length [cm].

	Coefficient	95% CI	P-value
Daytime SpO_2,_ %	−0.39	−0.71 to −0.07	0.017
Body height, cm	0.06	−0.14 to 0.26	0.543
Sex (female)	−2.13	−6.48 to 2.22	0.337
Subjective sleep quality^a^, %	−0.02	−0.06 to 0.02	0.290
Intercept	57.19	13.4 to 100.99	0.010

SpO_2_ = arterial oxygen saturation.

^a^ Subjective sleep quality was assessed by a 100-mm visual analogue scale, ranging from 0 “very bad” to 100 “very good.”

### Cognition

There were no altitude-dependent changes in the outcomes of PVT and TMT-A performance. However, the participants completed TMT-A within less time (i.e., had better performance) at 3,100 m vs. 760 m (Table 2).

## Discussion

In this observational study, 16 healthy participants living below 1,000 m have been exposed to 3,100 m for 3 weeks. The sudden hypobaric hypoxia led to hypoxemia, which recovered partially during the 3-week acclimatization period. Despite improvement in hypoxemia, the nocturnal SDB persisted for 3 weeks. Moreover, postural control remained impaired for over 2 weeks but normalized thereafter. No adverse acute or subacute altitude effects were observed in reaction time. These findings suggest that acclimatization at 3,100 m has differential effects on nocturnal oxygenation and control of breathing, postural control, and cognitive performance.

In a study of 51 healthy male lowlanders staying for 2 nights at 2,590 m, the nSpO_2_ improved on the second night compared to the first night at 2,590 m, and the numbers of nocturnal desaturations redirected towards baseline values (Latshang et al., 2013). Whereas in a study with 18 climbers at very high altitudes (4,497–6,865 m), nSpO_2_ normalized over time while periodic breathing increased during 2 weeks (Bloch et al., 2010). Additionally, in healthy individuals studied at an altitude of 4,559 m periodic breathing increased further compared to the first night (Nussbaumer-Ochsner et al., 2012).

In our study at 3,100 m, ODI, a surrogate for SDB, remained elevated during the acclimatization period of 3 weeks. However, when referring to the classifications of obstructive sleep apnea severity at low altitude (Kapur et al., 2017), the observed values of 5.7–9.1 events/hour in ODI and AHI at 3,100 m, would represent mild SDB, however, the participants did not suffer from clinical symptoms related to obstructive sleep apnea. Therefore, the clinical relevance of the sub-acutely persistent SDB remains unknown. Moreover, varying tendencies of SDB during a prolonged stay at different altitudes have been reported and may be related to changes in the sensitivity of the chemoreceptors to oxygen and carbon dioxide (Bloch et al., 2015; Javaheri and Dempsey, 2013). An increase in ventilation may increase the partial pressure of oxygen but lower the partial pressure of carbon dioxide, offsetting further hypoxic ventilatory stimulation. In our study, the breathing frequency remained unchanged with altitude, but information about the tidal volume was lacking.

Several authors showed the negative effect of simulated altitude on postural control in healthy individuals through chamber experiments (Wagner et al., 2011; Nordahl et al., 1998; Holness et al., 1982). Moreover, the postural control was impaired in 51 healthy males at a moderate altitude of 1,630 m (Stadelmann et al., 2015). Several previous studies (Mutschler et al., 2023; Nordahl et al., 1998) revealed a negative influence of acute hypobaric hypoxia on postural stability mainly in the anteroposterior direction, what we could confirm with this study. Presumably, hypoxia disturbs the fine interplay within the brain, neural afferents and motor output needed to maintain postural control. Indeed, in our multiple regression analysis the decreased daytime SpO_2_ was associated with an elongation of the COPL (Table 4), which has not been investigated intensively before.

Many travelers ascending from low altitude to high altitude report poor sleep quality during the first nights (Bloch et al., 2015). However, subjective sleep quality was not reduced in this study, although the participants noted more and longer awakenings on the first night at 3,100 m. Nonetheless, nocturnal oxygenation and breathing parameters showed no correlation with next-day postural control or cognitive performance.

The literature on cognitive and psychomotor functions at high altitudes reveals conflicting results–some reports indicate impairments, especially at very high altitudes (Yan, 2014; de Aquino Lemos et al., 2012; Kramer et al., 1993), others found no change (Luks et al., 1998; Latshang et al., 2013; Beaumont et al., 2007). Based on previous and current study findings, cognitive performance seems not to be uniformly impaired at altitudes between 3,000 and 3,500 m (Reiser et al., 2023; Pramsohler et al., 2017/05). The current findings suggest that cognitive performance does not further improve with better oxygenation during the acclimatization.

Little is known about sex differences during acute altitude exposure and acclimatization. We found that the female participants had fewer nocturnal oxygen desaturations, but otherwise, we found no sex-related differences (Table 3). Unfortunately, information about any hormonal contraceptives and menstrual cycle, which might have influenced our results, was not available.

This study has several limitations. The sample size may have attenuated other moderate effects in the acclimatization capacity when staying at 3,100 m. Potential sex differences and other influencing factors could not be conclusively evaluated. Furthermore, depending on time constants, some acclimatization processes might have escaped the measurements after one and 3 weeks. Since this study investigated the effect of 3 weeks at 3,100 m in healthy young participants, those findings should not be generalized to other populations, especially those with risk factors and comorbidities. For those vulnerable populations, further high-altitude studies are required.

## Conclusion

In healthy lowlanders staying at 3,100 m after rapid ascent, nSpO_2_ increased over 3 weeks but did not reach low altitude values. Postural control was impaired with acute exposure to altitude and remained impaired for more than 1 week despite improvements in hypoxemia, suggesting slower reversibility. Altitude did not affect vigilance and subjective sleep quality despite persistent sleep-disordered breathing. These acclimatization effects can guide future studies investigating the high-altitude acclimatization effects in elderly or patients with pre-existing diseases staying for holidays or rehabilitation purposes at high altitudes.

## Data Availability

The raw data supporting the conclusions of this article will be made available by the authors, without undue reservation.
